# Pelvic circumferential compression devices for prehospital management of suspected pelvic fractures: a rapid review and evidence summary for quality indicator evaluation

**DOI:** 10.1186/s13049-020-00762-5

**Published:** 2020-07-13

**Authors:** Robin Pap, Rachel McKeown, Craig Lockwood, Matthew Stephenson, Paul Simpson

**Affiliations:** 1 Joanna Briggs Institute, University of Adelaide, Adelaide, Australia; 2 School of Health Sciences, Western Sydney University, Sydney, Australia

**Keywords:** Pelvic fracture, Trauma, Prehospital care, Rapid review, Quality indicator

## Abstract

**Background:**

Pelvic fractures, especially when unstable, may cause significant haemorrhage. The early application of a pelvic circumferential compression device (PCCD) in patients with suspected pelvic fracture has established itself as best practice. Ambulance services conduct corresponding performance measurement. Quality indicators (QIs) are ideally based on high-quality evidence clearly demonstrating that the desirable effects outweigh the undesirable effects. In the absence of high-quality evidence, best available evidence should be combined with expert consensus.

**Objectives:**

The aim of the present study was to identify, appraise and summarize the best available evidence regarding PCCDs for the purpose of informing an expert panel tasked to evaluate the validity of the following QI: A patient with suspected pelvic fracture has a PCCD applied.

**Methods:**

A rapid review of four databases was conducted to identify relevant literature published up until 9 June 2020. Systematic reviews, experimental, quasi-experimental and observational analytic studies written in English were included. One author was responsible for study selection and quality appraisal. Data extraction using a priori extraction templates was verified by a second reviewer. Study details and key findings were summarized in tables.

**Results:**

A total of 13 studies were assessed to be eligible for inclusion in this rapid review. Of these, three were systematic reviews, one was a randomized clinical trial (crossover design), two were before-after studies, and seven were retrospective cohort studies. The systematic reviews included mostly observational studies and could therefore not be considered as high-level evidence. Overall, the identified evidence is of low quality and suggests that PCCD may provide temporary pelvic ring stabilization and haemorrhage control, although a potential for adverse effects exists.

**Conclusion:**

Given the low quality of the best available evidence, this evidence would need to be combined with expert consensus to evaluate the validity of a related quality indicator before its implementation.

## Background

Exsanguinating haemorrhage is one of the leading causes of death in patients suffering major trauma [1]. Besides catastrophic external haemorrhage, blood loss may occur from thoracic, abdominal, pelvic or limb injuries. Any of these alone or in combination can produce significant hypovolemia. Especially injury to the bony pelvis with disruption of the pelvic ring and damage to adjacent blood vessels may cause severe bleeding and can be associated with considerable morbidity and mortality [2–4]. As substantial force is required to cause fracture of the pelvic ring, some of the most frequent mechanisms of this injury involve road traffic accidents, falls from height and localized crush injuries [5, 6]. However, in the elderly with osteoporosis, disruption of the pelvic ring can also occur from low-energy mechanism [7]. Pelvic ring fractures may be classified in a number of ways. Most commonly, the Tile [8] and Young-Burgess [9] classification systems are used. These divide pelvic ring injuries into various types based on stability/instability of the posterior sacroiliac complex (Tile type A: stable, Tile type B: rotationally unstable, Tile type C: vertically and rotationally unstable) and vector of injuring force (lateral compression types, anterior-posterior types, vertical shear types and combined mechanisms) respectively. Considering the potentially life-threatening haemorrhage associated with pelvic ring fractures, rapid identification and management are critical to optimize patient outcomes.

Historically, prehospital management in the form of pelvic binding was performed when inspection and palpation of the pelvis revealed deformity, instability and pain. However, the diagnostic reliability of identifying a pelvic fracture by physical examination is questionable, particularly in the patient with decreased level of consciousness [10–12]. Furthermore, manipulating and especially springing the pelvis carries significant risk of disrupting any clot that may have formed and thus interfering with any spontaneous haemostasis [11]. Therefore, the decision to apply a pelvic circumferential compression device (PCCD) in any blunt trauma patient with *suspected* pelvic ring fracture based predominantly on the mechanism of injury and any visual signs such as bruising around the pelvis is increasingly being advocated as best practice in the prehospital care [13–15]. As the name implies, the intended purpose of a PCCD is to wrap around and stabilize the pelvic ring thereby limiting haemorrhage from cancellous bone or venous sources. The placement of a PCCD on a patient with a mechanism of injury suggestive of pelvic ring disruption is now commonly regarded to be an indicator of high-quality prehospital trauma care [13–15]. As such, many ambulance services utilize this quality indicator (QI) in the measurement of their clinical performance [16].

A QI is an explicitly defined and measurable aspect of health care services indicative of a desirable structure, process or outcome [17]. That is to say, there is evidence and/or consensus that the indicator can be used to quantify the quality of service provided, and thus monitor changes in quality over time [18]. This measurement provides a tool to identify unwarranted variation, facilitate data-driven improvement efforts and assess their impact. Systematically developed QIs are ideally based on scientific evidence. This may stem from rigorously developed guidelines [19, 20], but preferably ﻿is based directly upon high-quality scientific evidence such as ﻿thoroughly conducted (trial-based) empirical studies or robust systematic reviews and meta-analyses of randomized controlled trials (RCT) [17, 21]. In areas or disciplines where such evidence is scarce, it may be necessary to combine the best available evidence with expert consensus [17, 22]. Since the methodical review of underpinning evidence is fundamental to the systematic development of quality indicators, the expert consensus process should also be evidence-informed. ﻿The RAND/UCLA appropriateness method (RAM) is a formal group judgement process developed in the 1980s by the Research and Development (RAND) Corporation and the University of California, Los Angeles (UCLA) [23]. It combines expert opinion and scientific evidence in the form of systematic literature reviews by asking panellists to rate, discuss, and then re-rate statements.

However, this prominent advantage that RAM has over other consensus processes may also be a deterring factor. A systematic review is conducted to provide the expert panel with all pertinent information that will guide evidence-based decision-making [23]. Due to the rigorous methods applied when conducing full systematic reviews, they can take an extensive period of time to complete [24, 25]. This may be particularly problematic when multiple areas are being covered, there is high complexity in the topic, or both. Rapid reviews are a form of knowledge synthesis in which components of the systematic review process are simplified or omitted to produce information in a more timely manner [26]. As such, rapid reviews may offer a time- and resource-efficient alternative to modify RAM and prevent a potentially protracted and misaligned decision timeline. Although the rapid review approach has several inherent limitations, it may be a suitable compromise to facilitate swift synthesis of available evidence and adequately inform decisions in a RAM expert consensus process.

The aim of the present study was to apply rapid review methods to identify, appraise and summarize the best available evidence regarding PCCDs and in doing so provide an evidence summary to inform an expert panel tasked to validate the QI used for the measurement of prehospital trauma care quality. More specifically, this rapid review aimed to investigate current evidence for the effectiveness and safety of non-invasive PCCDs. This study forms part of a larger research project aimed at developing and testing prehospital care quality indicators for the Australian setting (https://www.aspireproject.net).

## Methods

### Preliminary work

As the initial part of the larger research project, a scoping review was conducted in accordance with Joanna Briggs Institute (JBI) methodology [16]. The scoping review’s purpose was to map the attributes of ‘quality’ in the context of prehospital care, to chart existing international prehospital care QIs and explore their development processes. Identified QIs were categorized as either system/organizational/non-clinical (domain A) or clinical (domain B). Within these two domains, several sub-domains were formed, including ‘trauma care’ (sub-domain B.6). QIs describing in one way or another the application of a PCCD in a patient with suspected pelvic fracture were identified in several included articles and aggregated into one single QI concisely describing the specific clinical intervention (Table 1). Furthermore, the QI was labelled as a process indicator according to Donabedian’s model, and as a QI primarily addressing ‘effectiveness’, one of the attributes of ‘quality’ mapped in the review.

**Table 1:** The aggregated quality indicator originating, amongst others, from the preliminary scoping review

***QI-B.6.2. A patient with suspected pelvic fracture has a pelvic circumferential compression device (PCCD) applied. (Process Effectiveness)***	

### Rapid review

#### Literature search strategy

Guided by the approaches to rapid reviews and evidence summaries by JBI and the World Health Organization (WHO) [27], a rapid systematic literature review was conducted to develop a summary of the best available evidence concerning the placement of a PCCD in the prehospital environment. Systematic searches of four electronic databases (the Cochrane Library, the JBI Database of Systematic Reviews, PubMed and CINAHL) were conducted on 9 June 2020. No date range filters were set but the search was limited to studies involving human participants and written in English. Due to the small number of systematic reviews identified, the search was expanded to include lower levels of evidence [28]. Nevertheless, observational descriptive studies, case series and case reports were excluded, as were non-systematic literature reviews. The full search strategy is available in Appendix S1.

#### Study selection

One author (RP) carried out the literature search, screened the results by title and abstracts using Covidence (Covidence, Melbourne VIC, Australia), and performed full-text review of shortlisted articles based on pre-defined inclusion criteria. The pre-defined inclusion criteria were based on the following population, intervention, comparison, outcome, context, study design (PICOCS) criteria:

- Population: Trauma patients with suspected or confirmed pelvic fracture(s)
- Intervention: Application of a PCCD
- Comparison: No intervention (or wrapping sheet)
- Outcomes: Clinical endpoints and/or adverse effects
- Context: Emergency trauma care
- Study designs: Systematic review, experimental and quasi-experimental studies, and observational analytical studies.

#### Quality appraisal

Following the search, studies selected for retrieval were assessed for internal validity using applicable JBI critical appraisal checklists [27]. This risk-of-bias assessment was performed by one author (RP). The quality threshold scores on respective checklists was 7 out of 11 for systematic reviews, 8 out of 13 for randomized control trials, 6 out of 9 for quasi-experimental studies and 7 out of 11 for cohort studies. These scores equated to a minimum quality threshold of 60% which was deemed to indicate sufficient quality for the research to be included in the review.

#### Data extraction and synthesis

Data were extracted by one author (RP) and verified by another (RM) using a standardized extraction template created a priori in Microsoft Excel for Mac 2019 (Microsoft Corp., Richmond, WA, USA). For systematic reviews, the following data were extracted: author(s), year of publication, number of studies included their designs, whether meta-analysis was performed and key findings. For primary research studies, following data were extracted: author(s), year of publication, study objectives and design, number of participants, participant characteristics, device(s), and key findings. Each systematic review and primary study was assigned a level of evidence in accordance with JBI [28].

## Results

### Search and critical appraisal results

**Fig. 1:**
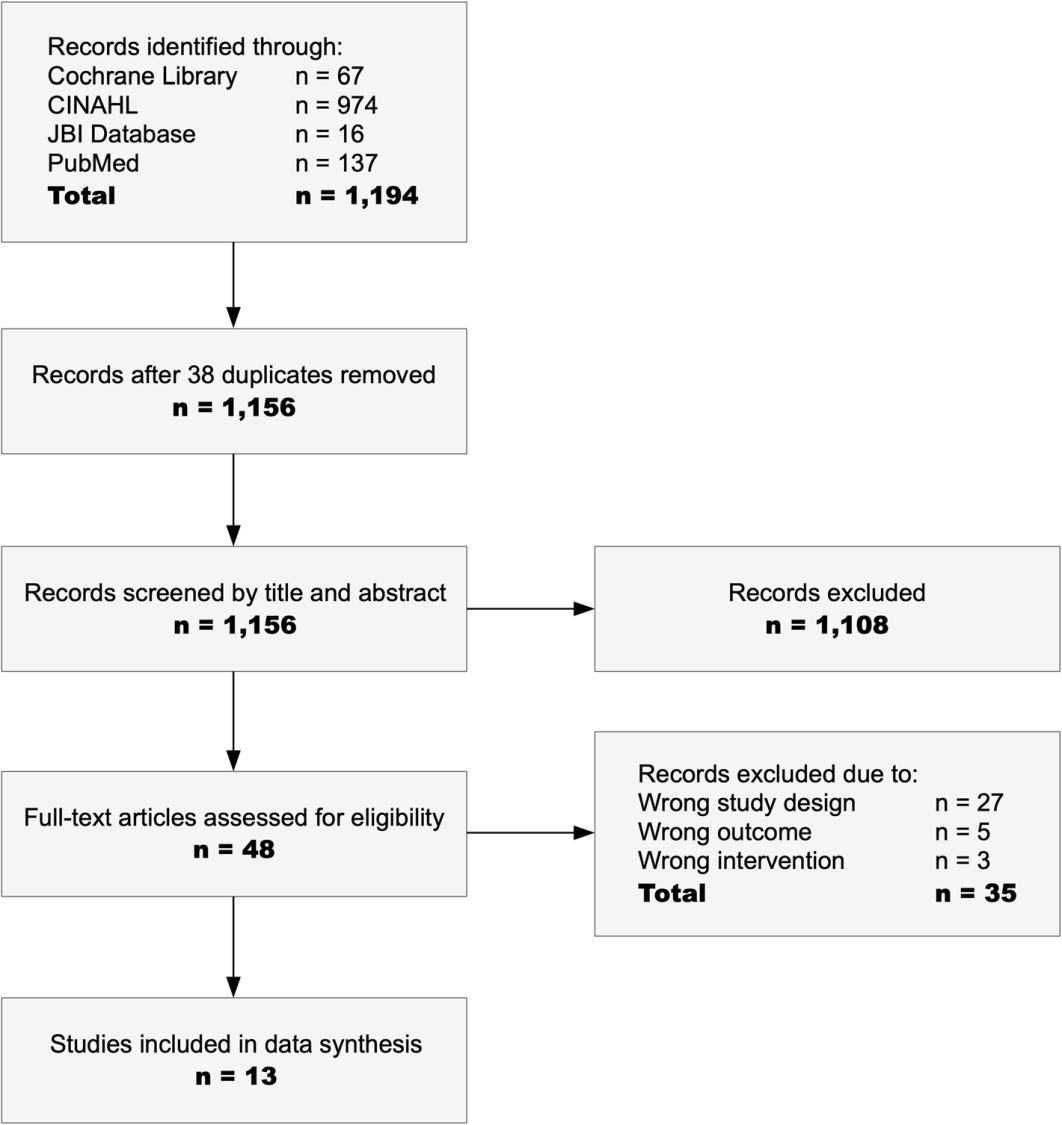
PRISMA flow chart of study inclusion

A total of 1194 potentially relevant records were identified through database searching (Fig. 1). Following the removal of 38 duplicates, 1156 records were retrieved for title and abstract screening. This found 1108 records to be incongruent with the inclusion criteria which were thus excluded and left 48 articles for full-text screening. Subsequently, 35 articles were excluded based on incompatibility with the review criteria which resulted in 13 articles being included for analysis in this rapid review. The 13 articles were critically appraised for methodological quality using applicable JBI critical appraisal tools. Based on the a priori minimum scores, all studies were included in this review.

### Description of the studies and characteristics of the evidence

Three systematic reviews [29–31], one randomized clinical trial (crossover design) [32], two before-after studies [33, 34], and seven retrospective cohort studies [35–41] were included (Tables 2 and 3). For systematic reviews, the level of evidence was assigned with consideration of included studies which addressed physiological effects and clinical outcomes such as reducing bleeding and decreasing mortality. Similar to the hierarchical rating of outcomes according to importance performed in the Grading of Recommendations Assessment, Development and Evaluation (GRADE) approach [42], these outcomes were considered most critical and thus given priority over other, less important outcome measures such as biomechanical effects in determining evidence level.

**Table 2:** Summary of included systematic reviews

Author	Year of Publication	Number of studies included	Study designs	Total number of patients/participants/cases	Meta-analysis performed	Summary	LOE^a^
Bakhshayesh, et al. [29]	2016	16	One RCT, two before-after studies, four retrospective cohort studies and nine case series (including six cadaver studies)	1377	No	Included studies suggest that PCCDs are effective in reducing a pelvic ring fracture. PCCDs may contribute to favourable physiological effects during the early phase of resuscitation. However, study results are inconclusive and conflicting with regards to other outcome measures, i.e. mortality, hospital length of stay, and intensive care unit (ICU) length of stay. Almost all types of PCCDs may potentially cause pressure ulcers if used for extensive periods due to inevitable tension over bony prominences.	2
Cullinane, et al. [30]	2011	6	One before-after study, two retrospective cohort studies, three case series (including two cadaver studies)	460	No	This systematic review was conducted for the development of clinical guidelines for surgical and non-surgical management of haemorrhage in pelvic fractures. Those studies which were included to evaluate the role of non-invasive temporary external fixation devices suggest that temporary binders reduce pelvic volume and may improve biomechanical stability. The effectiveness of non-invasive temporary external fixation devices limiting haemorrhage is unclear. They do not seem to affect mortality. Pelvic binders may cause tissue trauma due to shearing forces during the application process and skin breakdown over bony prominences when used over prolonged periods.	3
Spanjersberg, et al. [31]	2009	17	One before-after study, one retrospective cohort study, five case series (including three cadaver studies), seven case reports, three opinions	250	No	The reviewers concluded that available studies suggest that PCCDs may facilitate reduction of fractures and associated haemorrhage. However, data concerning mortality is lacking. Although the literature suggests no life-threatening complications occur with the use of PCCDs, the nature, severity and rates of complications is not fully known. Most obvious is a certain risk of damage to skin and potential iatrogenic injury to internal organs.	3

*LOE* Level of Evidence; *PCCD* Pelvic Circumferential Compression Device; *RCT* Randomized Clinical Trial; ^a^Based on included studies addressing physiological effects

**Table 3:** Summary of included primary clinical studies

Author	Year of publication	Study Design	Pertinent Objective(s)	Number of patients/ participants	Patients/participants and groups	Device(s)/Intervention(s)	Results summary	LOE
Schweigkofler, et al. [35]	2019	Retrospective Cohort study	To evaluate the effects of early (prehospital) application of a PCCD on transfusion requirements and mortality.	64	Trauma patients with Tile B (*n* = 31; 48.4%) and Tile C (*n* = 33; 51.6%) unstable pelvic fractures. A PCCD was applied prehospitally in 37 patients (58%); 27 (42%) received no prehospital pelvic binding.	Unspecified PCCD	There were higher ISS scores (29.7 vs 24.2) and lower probability of survival (RISC-II Prognosis 81% vs 89%) in patient who had a PCCD applied, however this was not statistically significant. There was also higher risk for massive transfusion (TASH-Scores 10% vs 6%) and average number of PRBC units transfused (10.5 vs 7.5) in patient with PCCD, again without statistical significance though. There was no statistically significance difference in mortality (20% vs 13.3% respectively).	3
Agri, et al. [36]	2017	Retrospective Cohort study	To describe the correlation between pelvic binders and patient outcomes.	228	﻿Adult (> 16 years) trauma patient with Tile A (*n* = 52; 22.8%), Tile B (*n* = 71; 31.1%) and Tile C (*n* = 105; 46.1%) pelvic fractures. Pelvic binders had been applied to in the field to 144 patients (63%) with comparable frequency among the three main fracture types (*p* = 0.61).	Unspecified PCCD (and AAE)	﻿Tile C fractures were associated with higher transfusion requirements (*p* < 0.0001) and higher mortality (*p* < 0.001). There was no statistically significant difference in injury severity between patient with PCCD and those without (ISS 26 vs 29; *p* = 0.99). Pelvic binders were not associated with differences in PRBC transfusion requirements (0 vs 2; *p* = 0.91) or mortality rates at 48 h (23% vs 18%; *p* = 0.5) or 30 days (25% vs 11%; *p* = 0.51) compared to the absence of pelvic binders. There were also no statistically significant differences in SBP, HR, SI, lactate level, SBD or need for AAE. No differences were detected in any of these variables even when selecting unstable fracture types (B1, B3 and C) only.	3
Hsu, et al. [37]	2017	Retrospective Cohort study	To compare the effects of early pelvic binding (based on suspicion of pelvic injury) with late pelvic binding (after fracture confirmation by radiography)	204	Trauma patients with a loss of consciousness or GCS < 13, SBP < 90 mmHg, fall from ≥6 m; injury to multiple vital organs, and suspected pelvic injury. Pelvic binders had been applied to 56 (27.5%) patients after confirmation of pelvic fracture and 148 (72.5%) patients with suspected pelvic injury.	SAM Pelvic Sling® II	There were no statistically significant differences in hospital LOS, ICU LOS, RTS, ISS score; percentage of SBP < 90 mmHg, GCS, percentage of AIS ≤3, angiography for AAE or mortality. However, those patients who received early pelvic binding had significantly less blood transfusion requirements (2462 ml vs 4385 ml; *p* = 0.009). Furthermore, uni- and multivariant regression analysis to adjust for confounders revealed significantly reduced mortality rates associated with early binding (*p* = 0.030 and *p* = 0.039 respectively).	3
Fu, et al. [38]	2013	Retrospective Cohort study	To evaluate the effects of PCCDs in patients with pelvic fractures who required transfer to trauma centres.	585	Patients with stable (*n* = 450; 76.9%) and unstable (*n* = 135; 23.1%) pelvic fractures who were transferred to a trauma centre within 24 h.	Unspecified PCCD or sheet wrapping	﻿The patients with stable pelvic fracture who received pretransfer PCCDs (*n* = 62; 13.8%) required significantly fewer blood transfusions (120.2 ml vs 231.8 mL; *p* = 0.018), had shorter intensive care unit LOS (1.7 days vs 3.4 days; *p* = 0.029) and shorter hospital LOS (6.8 days vs 10.4 days; *p* = 0.018) compared with patients who did not receive the pretransfer PCCD. The patients with unstable pelvic fractures who received pretransfer PCCDs (*n* = 91; 67.4%) also required significantly fewer blood transfusions (398.4 ml vs 1954.5 ml; *p* < 0.001), shorter intensive care unit LOS (6.6 days vs 11.8 days; *p* = 0.024) and shorter hospital LOS (9.4 days vs 19.5 days; *p* = 0.006) compared with patients who did not receive the pretransfer PCCD.	3
Pizanis, et al. [39]	2013	Retrospective Cohort study	To compare transfusion requirements of PRBC, LOS, mortality and incidence of lethal pelvic bleeding between patients which were treated by circumferential sheets, binders and c-clamps.	192	Trauma patients with fractures or disruptions of the pelvic ring. (The median age of patients treated with binders was significantly lower than in those treated with sheets of c-clamps.) One-hundred-and-thirty-three patients (69%) were treated with c-clamp, 31 (16%) with sheets and 28 (15%) with binders.	Unspecified PCCDs, sheet wrapping and c-clamp	There were no statistically significant differences in PRBC requirements (*p* = 0.26), LOS (*p* = 0.20) or mortality (*p* = 0.08). However, wrapping sheets were associated with a significantly higher incidence of lethal bleeding compared to PCCD and c-clamp (23% vs 4% vs 8%; *p* = 0.02).	3
Knops, et al. [32]	2011	Randomized controlled trial	To quantify the pressure at the region of the greater trochanters and the sacrum, induced by PCCDs in healthy individuals.	80	Healthy individuals lying on a spine board and lying on a hospital bed.	Pelvic Binder®, SAM-Sling® and T-POD®	Whilst lying on a spine board, the maximum pressure on the skin at the area of the greater trochanter exceeded 9.3 kPa (tissue damage threshold) with all three devices. No correlations of maximum pressure with BMI, waist size, or age on a spine board at the area of the greater trochanter were observed, except with an increase in maximum pressure with age (*p* = 0.031) when using one of the devices (SAM-Sling®). Whilst lying on the hospital bed, considerable reductions in maximum pressure, were found with all devices, in most cases below 9.3 kPa.	1
Tan, at al [33].	2010	Before-after study	To measure the immediate biomechanical and hemodynamic effects of pelvic binding.	15	Patients with unstable pelvic fractures who presented to the emergency department and who did not receive prehospital pelvic binding.	T-POD®	Application of the PCCD reduced pubic symphyseal diastasis by 60% (range 24–92%, *p* = 0.01). Mean values of mean arterial pressures increased significantly from 64.7 to 81.2 mmHg (*p* = 0.04). Similarly, heart rates decreased significantly from 106 to 93 beats per minute (p = 0.04).	2
Croce, et al. [40]	2007	Retrospective Cohort study	To compare the efficacy of pelvic binding to EPF.	186	Trauma patients with fractures or disruptions of the pelvic ring. Ninety-three patients (50%) were treated with EPF and 93 (50%) had the T-POD applied.	T-POD®	There were no differences in age or shock severity. Those patients who had a T-POD applied had significantly reduced 24-h (4.9 U vs 17.1 U; *p* < 0.0001) and 48-h transfusions (6.0 U vs 18.6 U; *p* < 0.0001). Compared to EPF, the T-POD also facilitated significantly decreased hospital LOS (16.5 days vs 24.4 days; *p* < 0.03). There was reduced mortality with the T-POD, however, this was not statistically significant (26% vs 37%; *p* = 0.11).	3
﻿Ghaemmaghami, et al. [41]	2007	Retrospective Cohort study	To assess the effectiveness of early application of a PCCD when compared to no device.	236	Patients with pelvic fractures and at least one of the following risk factors: - unstable fracture - age > 55 years - hypotension One-hundred-and-eighteen patients (50%) were treated with the PCCD and 118 (50%) did not receive any standardized pelvic binding other than occasional sheet wrapping.	Unspecified PCCD	The two groups had similar fracture patterns, age, and injury severity. In the comparison of patients wo were treated with a PCCD with those who received no standardized pelvic binding, there were no significant differences in mortality (23% vs 23%; *p* = 0.92), need for AAE (11% vs 15%; *p* = 0.35), or 24-h transfusion (5.2 U vs 4.6 U; *p* = 0.64).	3
Krieg, et al. [34]	2005	Before-after study	To assess the effectiveness of a PCCD in reducing and stabilizing pelvic ring fractures.	13	Adult patients (> 16 years) with partially stable or unstable pelvic fractures with external or internal rotation pattern.	Unspecified PCCD	In patients with external rotation, the PCCD significantly reduced the pelvic width by 9.9 ± 6.0%. In patient with internal rotation, there was no significant over-pressurization due to application of the PCCD.	2

*AAE* Arterial Angio-Embolization; *AIS* Abbreviate Injury Score; *BMI* Body Mass Index; *EPF* External Pelvic Fixation; *GCS* Glasgow Coma Score; *HR* Heart Rate; *ICU* Intensive Care Unit; *ISS* Injury Severity Scale; *LOE* Level of Evidence; *LOS* Length of Stay; *PCCD* Pelvic Circumferential Compression Device; *PRBC* Packed Red Blood Cells; *RISC* Revised Injury Severity Classification; *RTS* Revised Trauma Score; *SBD* Standard Base Deficit; *SBP* Systolic Blood Pressure; *SI* Shock Index; *TASH* Trauma Associated Severe Haemorrhage

### Summary of the evidence and clinical bottom line

Tables 2 and 3 provide summaries of the included studies’ findings. Generally, the evidence in support of the application of a PCCD in a patient with suspected or confirmed pelvic fracture is weak. Whilst three systematic reviews were identified, the design of included studies (mostly observational) in these reviews lowered their level of evidence. None of the systematic reviews included a meta-analysis of included studies. Bakhshayesh, et al. (2016) [29] explicitly stated that it was not possible to combine results due to heterogeneity amongst included studies. This heterogeneity is echoed in the primary clinical studies identified in this rapid review making synthesis of results challenging. Furthermore, the limited clinical research is comprised predominantly of historical cohort studies, which induces inherent and considerable risk of bias.

Included studies which address the biomechanical effects of PCCDs indicate the devices facilitate a reduction in pelvic volume and improvement in biomechanical stability [29–31, 33, 34]. Of the included studies, several suggest that PCCDs, especially if applied early, may contribute to a variety of desirable physiological effects [29–31, 33, 37, 38, 40]. Yet, results concerning other, more critical outcome measures such as mortality and hospital or intensive care unit length of stay are ambivalent or conflicting [29–31, 35–37, 39, 41]. Three studies included sheet wrapping as an improvised method to stabilize the pelvic ring [38, 39, 41]. However, only one of these (Pizanis, at al. 2013) [39] compared this method to the application of a commercial PCCD and demonstrated benefits in using a PCCD over improvised pelvic binding in reducing mortality. The systemic reviews consistently report on potential adverse effects of PCCDs. These including mostly skin damage, myonecrosis and peroneal nerve palsy when used for extended periods of time, but also injury to internal organs as a result of shearing forces during the application process [29–31].

The clinical bottom line is that there is no high-level evidence that the application of a PCCD reduces haemorrhage or mortality in suspected or confirmed pelvic fractures. The best available evidence suggests that a ﻿PCCD provides temporary pelvic ring stabilization and can serve as an adjunct to early haemorrhage control. The application of PCCD carries a certain potential for iatrogenic harm, however, clinical benefits seem to outweigh this risk. Given the limited data to show undisputable benefit, further research on this topic is needed. In particular, there is a lack of research in the prehospital arena as well as studies which examine the effectiveness and safety of PCCDs in specific pelvic fractures types according to Young-Burgess classification as this mechanistic classification is more practical for the prehospital context.

## Discussion

﻿Patients suffering pelvic fractures are at risk of severe and potentially life-threatening bleeding [43, 44]. Especially patients with unstable pelvic fracture types are at high risk of exsanguinating haemorrhage [45, 46]. Palpation of the pelvis is unreliable in detecting instability and has been associated with dislodging clots and initiating further blood loss [47]. Therefore, in early major trauma care, the presence of pelvic disruption should be based on suspicion after consideration of the mechanism of injury rather than confirmation by physical examination. PCCDs have been shown to provide effective biomechanical ﻿reduction in partially stable and unstable pelvic fractures [48]. A clinically reasonable assumption is that the prompt application of a PCCD facilitates early stabilization of unstable fractures and thus leads to favourable physiological effects and ultimately desirable patient outcomes. This rapid review aimed to summarize current evidence for the effectiveness and safety of non-invasive PCCDs and identified several, albeit methodologically weak studies in support of the intervention. As such, this rapid review was unable to identify high-quality evidence and the best available evidence should be combined with expert consensus in a process such as RAM to assess the validity of the QI under discussion.

Health care quality measurement and improvement are complex endeavours. Considering the resources health care organizations invest in them and the potential adverse consequences if conducted poorly [49, 50], it is important to get it right from the start. Unfortunately, indicators are often chosen because the required data is easily attainable rather than because they are evidence-based [51]. When indicators are developed or transferred between health care systems, it is critical to review their supporting evidence and the quality thereof [52, 53]. A QI is preferably based on high-quality evidence clearly demonstrating that the desirable effects outweigh the undesirable effects. Such evidence is produced by large, thoroughly conducted RCTs that demonstrate consistent impressive benefits with limited adverse effects and minimal cost. In the absence of such high-quality evidence, best available evidence should be combined with expert consensus to assess the validity of the indicator. Therein lies the essence of a *quality* indicator and what distinguishes it from a *performance* indicator – a QI has scientific credibility, i.e. there is evidence and/or expert consensus that the indicator can be used to make a judgement about quality [17]. Not only are health care quality improvement managers increasingly required to deploy such scientific methods to develop measures of quality, but also they are required to do so in limited amounts of time [54]. This presents a potential misalignment between QI development and timelines set by organizational quality improvement needs [55, 56]. This paper presents an example of a fast-tracked systematic literature review methodology which balanced its scope against time and resource constraints, and in doing so may prevent protraction and provide a timely evidence summary to inform QI development. From inception to completion this rapid review took approximately 3 months; a relatively short timeframe compared to full systematic reviews which commonly take 12 to 24 months to complete [57, 58].

There are several significant limitations that the omission or simplification of systematic review methods induce. The search strategy was limited by restricting the number of databases consulted, excluding all non-English language papers, using more specific search terms and excluding lower levels of evidence. Databases were restricted in line with guidance for rapid reviews and evidence summaries by JBI. Whilst systematic reviewer and meta-analysts should conduct exhaustive searches in multiple databases, rapid reviews commonly omit several databases to focus on those expected to yield best results. This approach is justifiable by studies which have demonstrated only marginal improvement in relevant results by increasing the number of databases searched [59, 60]. The search for studies in rigorously conducted systematic reviews should not be restricted by language. Limiting results to those written in English inevitable introduces English language bias or Tower of Babel bias potentially leading to an over- or underestimation of an intervention’s effectiveness [61]. Reliable translation services, however, require time and financial resources making them a less suitable part of a rapid review search strategy. Optimal search strategies aim for maximum number of relevant references with minimal noise, i.e. best sensitivity and specificity. In this balance, rapid reviews commonly lean towards specificity. The search terms in this rapid review were more specific by using narrower MeSH terms (e.g. MH “pelvic fractures”), using Boolean operators to narrow MeSH headings (e.g. (pelvic bones [mh] OR pelvis [mh]) AND (fractures, bone [mh] OR wounds and injuries [mh]) and by avoiding less common keywords (e.g. splint). JBI evidence summaries are ideally based on several systematic reviews, however, when no systematic reviews are identified, lower levels of evidence are included [27]. This rapid review adopted the approach but leaned towards more comprehensive inclusion by lowering the methodological exclusion threshold to observational descriptive studies. Whilst data extraction was verified by a second reviewer, the preceding study selection and quality appraisal was performed by only one reviewer. Expediting the review process in this way is frequently done in rapid reviews, however, introduces considerable risk of bias and error.

## Conclusion

This study provides an example of how the timely knowledge synthesis through the deployment of a streamlined rapid review approach can inform QI development. More specifically, the study has reviewed best available evidence regarding the application of a PCCD in patients with suspected pelvic fractures and summarized this into a synopsis for feasible consideration by an expert panel tasked to assess the validity of a related QI. The process of applying a PCCD is not clearly linked to desirable clinical outcomes and does carry a potential for iatrogenic harm. Nevertheless, the clinical benefits seem to outweigh risks. This best available evidence is of low quality strengthening the need for its perusal by an expert panel before possible QI implementation.

## Supplementary information

**Additional file 1.**

## Data Availability

The datasets used and/or analysed during the current study are available from the corresponding author on reasonable request.

## Abbreviations

AAE: Arterial Angio-Embolization
AIS: Abbreviate Injury Score
BMI: Body Mass Index
CINAHL: Cumulative Index to Nursing and Allied Health Literature
EPF: External Pelvic Fixation
GCS: Glasgow Coma Score
GRADE: Grading of Recommendations Assessment, Development and Evaluation
HR: Heart Rate
ICU: Intensive Care Unit
ISS: Injury Severity Scale
JBI: Joanna Briggs Institute
LOE: Level of Evidence
LOS: Length of Stay
PCCD: Pelvic Circumferential Compression Device
PICOCS: Population, Intervention, Comparison, Outcome, Context, Study design
QI: Quality Indicator
RAM: RAND/UCLA Appropriateness Method
RCT: Randomized Controlled Trial
RISC: Revised Injury Severity Classification
RTS: Revised Trauma Score
SBD: Standard Base Deficit
SBP: Systolic Blood Pressure
SI: Shock Index
TASH: Trauma Associated Severe Haemorrhage
WHO: World Health Organization
