# Supplementary material for: Pelvic circumferential compression devices for prehospital management of suspected pelvic fractures: a rapid review and evidence summary for quality indicator evaluation
Source: Scand J Trauma Resusc Emerg Med. 2020 Jul 13;28:65. doi: 10.1186/s13049-020-00762-5 (PMC7359240; doi:10.1186/s13049-020-00762-5)
Supplement: Supplementary file 1 — Additional file 1. [file 13049_2020_762_MOESM1_ESM.docx]

**Appendix 1**

Search Strategy for **PubMed**

| **Search** | **Search Terms** |
| --- | --- |
| #1 | (pelvic bones[mh] OR pelvis[mh]) AND (fractures, bone[mh] OR wounds and injuries[mh]) |
| #2 | pelvi*[tw] AND fractur*[tw] |
| #3 | bind*[tw] OR t-pod[tw] OR tpod[tw] OR wrap[tw] OR circumferential compression[tw] OR sling[tw] OR sheet[tw] OR non-invasive[tw] |
| #4 | (#1 OR #2) AND #3 |

Search Strategy for **CINAHL**

| **Search** | **Search Terms** |
| --- | --- |
| #1 | MH ”pelvic fractures” OR (MH ”pelvic bones” AND MH ”fractures”) |
| #2 | TX pelvi* AND TX fractur* |
| #3 | TX bind* OR TX t-pod OR TX tpod OR TX wrap OR TX circumferential compression OR TX sling OR TX sheet OR TX non-invasive |
| #4 | (#1 OR #2) AND #3 |

Search Strategy for **Cochrane Library**

| **Search** | **Search Terms** |
| --- | --- |
| #1 | MeSH descriptor: [pelvic bones] this term only |
| #2 | MeSH descriptor: [pelvis] this term only |
| #3 | MeSH descriptor: [fractures, bone] this term only |
| #4 | MeSH descriptor: [wounds and injuries] this term only |
| #5 | (#1 OR #2) AND (#3 OR #4) |
| #6 | pelvi* AND fractur* |
| #7 | bind* OR t-pod OR tpod OR wrap OR circumferential compression OR sling OR sheet OR non-invasive |
| #8 | (#5 OR #6) AND #7 |

Search Strategy for **JBI Database**

| **Search** | **Search Terms** |
| --- | --- |
| #1 | pelvi* AND fractur* |
| #2 | bind* OR t-pod OR tpod OR wrap OR circumferential compression OR sling OR sheet OR non-invasive |
| #3 | #1 AND #2 |
